# Head and mandible shapes are highly integrated yet represent two distinct modules within and among worker subcastes of the ant genus *Pheidole*

**DOI:** 10.1002/ece3.7422

**Published:** 2021-05-01

**Authors:** Alexandre Casadei‐Ferreira, Nicholas R. Friedman, Evan P. Economo, Marcio R. Pie, Rodrigo M. Feitosa

**Affiliations:** ^1^ Departamento de Zoologia Universidade Federal do Paraná Curitiba Brazil; ^2^ Biodiversity and Biocomplexity Unit Okinawa Institute of Science and Technology Graduate University Onna Japan

**Keywords:** 3D, ant, geometric morphometrics, microCT, New World

## Abstract

Ants use their mandibles for a wide variety of tasks related to substrate manipulation, brood transport, food processing, and colony defense. Due to constraints involved in colony upkeep, ants evolved a remarkable diversity of mandibular forms, often related to specific roles such as specialized hunting and seed milling. Considering these varied functional demands, we focused on understanding how the mandible and head shape vary within and between *Pheidole* subcastes. Using x‐ray microtomography and 3D geometric morphometrics, we tested whether these structures are integrated and modular, and how ecological predictors influenced these features. Our results showed that mandible and head shape of majors and minor workers tend to vary from robust to slender, with some more complex changes related to the mandibular base. Additionally, we found that head and mandible shapes are characterized by a high degree of integration, but with little correlation with feeding and nesting habits. Our results suggest that a combination of structural (allometric) constraints and the behavioral flexibility conferred by subcaste dimorphism might largely buffer selective pressures that would otherwise lead to a fine‐tuning between ecological conditions and morphological adaptation.

## Graphical abstract

In this manuscript, we used X‐ray microtomography and 3D geometric morphometrics, to test whether head and mandibles in workers of the ant genus *Pheidole* are integrated and modular, and how ecological predictors influenced these features. Our results suggest that a combination of structural (allometric) constraints and the behavioral flexibility conferred by subcaste dimorphism might largely buffer selective pressures that would otherwise lead to a fine‐tuning between ecological conditions and morphological adaptation.

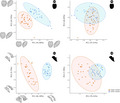

## 1 INTRODUCTION

Morphology plays a major role in mediating the interactions of an organism with its environment (*e*.*g*., Marques et al., 2019). Feeding, mating, and dispersal are some of these crucial tasks in which fitness can be affected by morphological variation (Chapman, 2013). In social insects, the link between morphology and task performance is amplified by the separation between reproductive and nonreproductive castes (Peeters & Ito, 2001). The reproductive caste in a social insect colony (queens and males) has as its primary role mating and dispersal, in which the queens’ abdomens bear well‐developed ovaries for egg production, and the mesosoma, of queens and males, house strong muscles and wings for dispersal (Peeters & Ito, 2001). The nonreproductive caste (workers), which is associated with feeding and colony maintenance, in general, has no constraints related to mating and dispersal, which allows independence in morphological modifications from a trade‐off with those reproductive pressures. In this sense, the worker caste can further specialize into morphologically distinct forms and can differ in size or shape. However, they are constrained by the behavioral and functional demands for the ergonomic efficiency of the colony, such as prey capture, defense, food storage, and processing (Wilson, 1984; Pie & Traniello, 2007; Pie & Tschá, 2013; Powell, 2008, 2009; Powell & Franks, 2005, 2006; Oster & Wilson, 1978; Mertl & Traniello, 2009; Traniello, 2010, Tschá & Pie, 2019).

Among the morphological modifications associated with colony functions in social insects, the mandible is a fundamental and highly specialized appendage. While this tool is crucial for many insects, ant mandibles are remarkably modified and extremely versatile, more than in any other group (*e*.*g*., Barden et al., 2017; Larabee et al., 2018; Lattke et al., 2018). Ants use their mandibles to carry out a wide diversity of tasks strongly related to substrate manipulation, brood transport, food processing, and defense. Despite the limitation of having to support many basic colony functions, ants have evolved an extraordinary diversity of mandibular forms, often related to functions such as specialized hunting (*e*.*g*., Larabee & Suarez, 2014) and seed milling (*e*.*g*., Moffett, 1985; Bernadou et al., 2016). These modifications and the mandibles themselves should not be understood as isolated features, but rather as components of ant head shape. In this sense, ant heads bear the muscles associated with mandibles, facilitating the abduction and adduction movements, and absorbing all the strength required by this apparatus (Richter et al., 2019). However, the functional significance of different shape variants and ecological pressures driving mandible evolution are poorly understood.

Mandibles are true multifunctional tools, important for diverse tasks necessary for feeding, building, and social care. Among predatory ants (*i*.*e*., Amblyoponinae, Dorylinae, Ponerinae, Ectatomminae, and some Myrmicinae), mandibles are crucial for hunting success, since they are commonly used as a grasping apparatus to subdue the prey until the ant can sting it (*e*.*g*., Gronenberg, 1996; Dejean & Evraerts, 1997; Gronenberg et al., 1998; Dejean et al., 1999; Schatz & Wcislo, 1999; Ward & Fisher, 2016). In addition to grasping, ants also use their mandibles to transport and process food items (*e*.*g*., dismember, grind, puncture, and tear their food) (Hölldobler, 1985; Gissel Nielsen, 2001; Schöning et al., 2005; Czaczkes et al., 2011; Czaczkes & Ratnieks, 2013; Bernadou et al., 2016). Although ants are often considered generalists and scavengers, several ant groups exhibit narrow diets, such as specialist predators, fungus farmers, and seed harvesters (*e*.*g*., Brown et al., 1979; Mueller et al., 2001; Larabee & Suarez, 2014).

In seed harvester ants, seeds are collected by foraging workers (*i*.*e*., slender body and mandibles). These workers then bring the seeds into the nest where they can be stored. Later, larger workers with specialized phenotypes (*i*.*e*., robust body and mandibles) can process them. Such species often inhabit desert and savanna ecosystems (Brown et al., 1979) but can also be found in tropical forests (Wilson, 2003). Among the several ant genera that exhibit this behavior, *Pheidole* Westwood (Myrmicinae: Attini) can be considered an ideal study system due to its inordinate diversity, wide distribution, and the presence of a specialized subcaste.

*Pheidole* is the most diverse genus among ants, with 1,167 extant species (Bolton, 2020). However, estimates suggest the existence of at least 1,500 *Pheidole* species (Wilson, 2003) and possibly well over 2,000 (Economo et al., 2019). One of the most remarkable peculiarities of *Pheidole* is the conspicuous dimorphism between its workers. This dimorphism is characterized by the division between "minor workers" and more robust and macrocephalic workers, known as "major workers" or "soldiers" (Wilson, 2003). Both subcastes also have morphological modifications involving the reduction of the sting apparatus and the absence of functional ovaries. The sting apparatus in *Pheidole* is quite atrophied (Kugler, 1978) and is likely to be nonfunctional. Along with sting simplification, the absence of functional ovaries (Hölldobler & Wilson, 2009) can result in a lower body volume, decreasing the energy cost involved in worker production for the colony, which allows for a large number of individuals coexisting in the nest (Hölldobler & Wilson, 2009), and the potential caste specialization related with sterility (Oster & Wilson, 1978). *Pheidole* majors are morphologically and behaviorally adapted in the nest and resources defense, food transportation and processing, as well as food storage (Mertl et al., 2010; Mertl & Traniello, 2009; Sempo & Detrain, 2004; Wilson, 1984, 2003). Pie and Traniello (2007) showed that morphological variation in majors and minor workers can be attributed mainly to allometric changes, with little dissociation between morphological features. Additionally, the authors demonstrated a lower degree of morphological integration in major workers than in minor workers, which was also found by Friedman et al., (2019) using different approaches for both measurements and analysis.

Although most *Pheidole* species are associated with generalist and scavenger habits, seed predation and harvesting account for a significant portion of their diet and have evolved independently multiple times in New and Old World species (Economo et al., 2015; Moreau, 2008). Macrocephaly is often correlated with seed processing in ants, and therefore with the demand for considerable muscle strength (Ferster et al., 2006). However, recent studies have questioned the relationship between worker head size and granivory in *Pheidole*. Holley et al., (2016) studied this relationship, predicting that majors in seed‐harvesting species should have the musculature optimized to open the seed, which would produce wider heads compared with those that do not consume seeds. Nevertheless, the authors found that *Pheidole* species did not exhibit a relationship between cephalic size and seed‐milling behavior. In contrast, Holley et al., (2016) reported that there is a greater difference in head size between major and minor workers in the granivorous species when compared to nongranivorous ones. However, these previous studies are based on 2D or linear morphometrics. The recently developed 3D approaches made it possible to capture almost all the shape variation, consisting of the most powerful tool to test for these issues. These approaches are especially useful when considering structures that are difficult to quantify, given their irregular surfaces (*e*.*g*., insect mandibles and mesosoma).

Considering the morphological complexity in *Pheidole*, we used x‐ray microtomography and 3D geometric morphometrics to investigate how mandible and head shape vary within and between its subcastes. Also, we tested whether these parts are integrated and modular, how much the allometric effect is responsible for differences in shape, and how ecological predictors influenced these features.

Due to the worker's conspicuous dimorphism, mainly related to size, as already pointed out by Pie and Traniello (2007), we predicted a strongly allometric effect on the head and mandible shape for majors and minors, following the pattern found for the genus. Because of the important role of the head in bearing the muscles associated with mandibles, we hypothesized that these structures (head and mandibles) are strongly integrated and should be part of the same module, with less covariation between modules than within them. In this scenario, the shape of head and mandible would covariate jointly, as the mandibular demands would be met by the shape of the head, thus optimizing its functions. However, some alternatives are possible, for instance, the lack of integration and modularity between head and mandible, as well as the indication of strong integration with most covariation occurring between modules instead of within them.

The lack of head and mandible integration and modularity in *Pheidole* would be possible if the mandibular demands do not depend directly on the head shape for the muscular volume and disposition. Thus, the mandible could have evolved as a structure semi‐independent from the rest of the head, which would result in a great plasticity and diversity of shapes. Additionally, it can indicate that generalized head shapes would allow different mandible forms to meet their demands. As demonstrated by Holley et al. (2016), the massive head volume in *Pheidole* major workers is not directly associated with pressures involving the requirement for a strong capacity for process seeds (*i*.*e*., mechanical function), thus, suggesting the possible dissociation between mandible and head shape. The second possibility is that these structures are strongly integrated, however, that they are not part of the same module. Considering the integration without modularity (Roseman et al., 2009), the genetic effects would not be strongly grouped as expected for structures that display significant modularity; instead, they may be spatially restricted but continuous, even overlapped (Zelditch et al., 2012). This would allow mandible and head to vary their shape similarly, without being affected as a single unit in its developmental and evolutionary process.

Several studies have pointed out that lifestyles (*e*.*g*., fossorial, marine, and parasitic) and feeding ecologies may be the main predictors for diversification and specialization of forms in different organisms (Da Silva et al., 2018; Olsen, 2017). Indeed, insects have mouthparts with characteristic adaptations that evolved in a context mainly related to the variety of exploitable food sources, which resulted in feeding specialization for better functional performance in many lineages (Krenn, 2019). To optimize mandible and head shape to explore their environment while considering the potential impact that ecological predictors play in morphological changes, we expect that feeding and nesting preferences should affect morphological patterns of these structures in *Pheidole* workers. However, an alternative possibility is that workers' dimorphism, as well as the behavioral flexibility they provide for the worker force, can reduce ecological pressures on head and mandible shape, which could enable these structures to vary independently of feeding and nesting habits.

We envision three contrasting scenarios of morphospace occupation for minor and major workers. Because majors tend to be behaviorally specialized for food processing and defense, we expect that head and mandible shape in these individuals would be strongly constrained to a set of optimal morphological optima when compared to minors. This would lead to a more restricted morphospace into which major workers could diversify, whereas minors would be freer to evolve different forms. Alternatively, given that minors are responsible for most quotidian colony functions, such as brood care, foraging, and nest maintenance, they would be more constrained by the need to perform simultaneously such a variety of tasks, whereas major workers could be freer to evolve into more specialized morphologies. Finally, major and minor morphologies could be so intimately linked in their developmental pathways that they would constrain one another in their evolutionary possibilities, such that their morphospace occupation would essentially mirror one another.

## 2 MATERIAL AND METHODS

We compiled a dataset of 3D models of major and minor workers (one specimen per subcaste) of a total of 27 *Pheidole* species (*N* = 54 specimens) (Table S1). We assumed that the morphological variation between individuals of the same subcastes within the same species is relatively small when compared to the variation between different subcastes and different species. We selected our focal species based on the conspicuous morphological variation among them, as well as the availability of material and the reliability of the associated biological data. However, as no molecular data are available for most of the selected species, explicit phylogenetic comparative analyses were not performed.

Our dataset represents the main groups recognized in the New World, where they cover a broad geographical distribution from the South of the United States to the South of Brazil. Additionally, they represent the main feeding and nesting habits found in *Pheidole*. We categorized their feeding behavior as granivorous and nongranivorous, and their nesting habits as soil, twigs, and plants. We chose to include species with missing biological data so that we could build a morphospace encompassing the largest possible morphological variation in the genus.

A micro‐CT/μCT scans ZEISS Xradia 510 Versa (Carl Zeiss AG) generated the scans used to construct those 3D models. Scan settings were selected according to yield optimum scan quality: 4x objective, exposure times between 1 and 5 s, source‐filter “Air,” voltage between 30 and 50 keV, power between 4 and 5W, and field mode “normal.” The combination of voltage, power, and exposure time was set to yield intensity levels between 15,000 and 17,000 across the whole specimen. Scan times varied from 27 to 50 min, depending on exposure times. Full 360‐degree rotations were done with a number of 801 projections. The resulting scans have resolutions of 1013 × 992 × 999 (H × W × D) pixels and voxel sizes range between 2.25 and 5.39 μm. The processing and postprocessing of raw DICOM data were performed with Itk‐snap 3.8.0 (Yushkevich et al., 2006). Desired volume renderings were generated by adjusting the range of color space to a minimum so that the outer surface of specimens remains visible at the highest available quality. The 3D models were rotated and manipulated to allow a complete virtual examination of scanned specimens.

**FIGURE 1:**
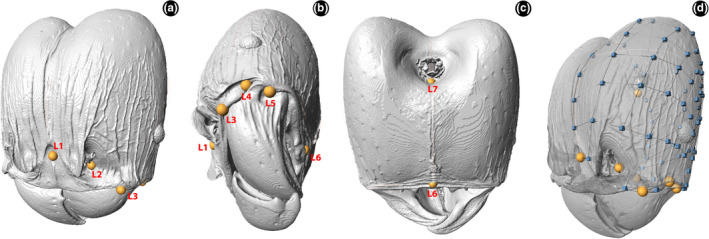
Position of landmarks (yellow dots) and semilandmarks (blue dots) on the head of major of *Pheidole* workers. (a) fronto‐oblique view; (b) antero‐oblique view; (c) ventral view; (d) transparency showing the patch and curves

We used Stratovan Checkpoint v. 2019.03.04.1102 (Stratovan Corporation [Software]) to measure the head shape by digitizing seven landmarks and 45 semilandmarks. The landmarks corresponded to the posterior clypeal midpoint (L1); tentorial pit (L2); anterior clypeal condyle (L3); posterior clypeal condyle (L4); head projection between atala and ventral condyle (L5); hypostomal margin midpoint (L6); and ventral nuchal midpoint (L7) (Figure 1a–c). Semilandmarks were divided into three curves and one patch, such that one curve started in the L7 and ended in L1; second, it started in L7 and ended in L6; the third started in L6 and ended in the L5 (Figure 1d). Lastly, the patch was placed connecting the four anchor points in L1, L5, L6, and L7 (Figure 1d). We opted to apply the measurements to a single half of the head due to bilateral symmetry.

**FIGURE 2:**
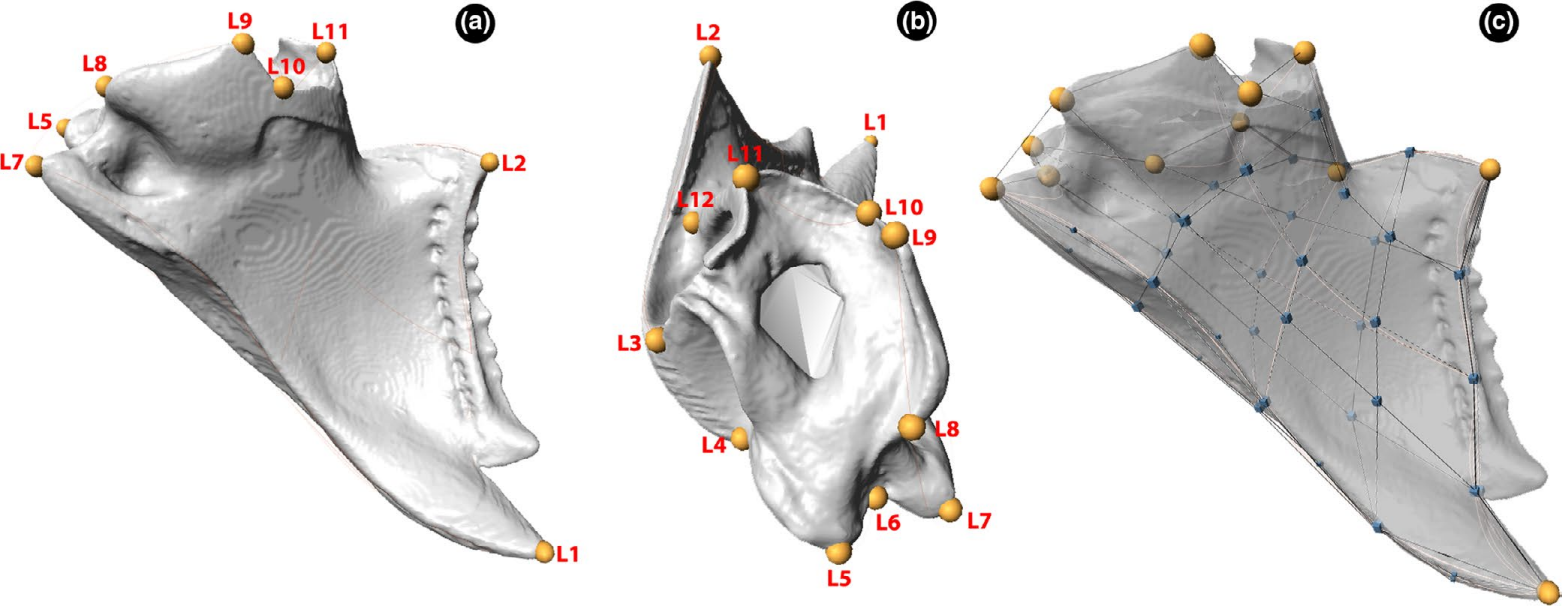
Position of landmarks (yellow dots) and semilandmarks (blue dots) on the mandible of *Pheidole* workers. (a) ventral view; (b) dorsal view; (c) transparency showing the patches and curves

We measured mandible shape by digitizing 12 landmarks and 41 semilandmarks using the same software. The landmarks corresponded to the apical tooth (L1); basal tooth (L2); anterior acetabulum (L3); posterior acetabulum (L4); atala (L5); notch between atala and ventral condyle (L6); ventral condyle (L7); ventrobasal corner (L8); dorsal‐basal corner (L9); ventral apodeme insertion corner (L10); dorsal apodeme insertion corner (L11); and anterior trulleum corner (L12) (Figure 2a, b). Semilandmarks were divided into five curves and two patches, in which one curve started in the L2 and ended in L3; the second and the third started in L4 and ended in L1, both extending in the external margin, one between the dorsal surface and the external margin, and the other between the ventral surface and the external margin; and the last two curves started in the L2 and ended in L1, one of these in the external margin e the other under the internal margin (Figure 2c). The patches were placed one in the dorsal and other in the ventral surface, in which the external was connected with the L1, L2, L3, and L5, and the external with L1, L2, L5, and L11 (Figure 2c).

All geometric morphometrics analyses were performed in R (R Development Core Team, version 3.6.2. 2019) using the geomorph package v. 3.3.2 (Adams et al., 2020). Landmark and semilandmark coordinates were aligned using Generalized Procrustes Analysis (GPA), in which the specimens were translated to a common location, scaled them to unit centroid size, and optimally rotated them using the least‐squares criterion (Rohlf & Slice, 1990). To test the influence of allometry, we used a Procrustes regression (Goodall, 1991; Adams & Collyer, 2018) of independent contrasts of shape (Procrustes coordinates) on independent contrasts of size (centroid size). Allometry was tested within both subcastes combined in a single dataset and for each subcaste separately. Statistical significance was assessed using a residual randomization permutation procedure (1,000 permutations). Additionally, we performed a Procrustes regression to access variation in allometric slopes between subcastes, testing common or unique allometries. To visualize shape changes associated with the allometric variation, we estimated the average allometric trends within groups (*i*.*e*., subcastes) using the common allometric component (CAC; Mitteroecker et al. 2004) approach.

Principal component analyses (PCAs) were performed to visualize the distribution of species shape along different axes in tangent space for majors and minor workers combined, as well as for each subcaste separately. Our discussion will focus on the axes that together explain at least 70% of the total variation. Thin‐plate spline deformation grids (Bookstein, 1991) were employed to visually describe the shape differences. To test the morphological integration between head and mandible, we ran a two‐block partial least squares analysis for Procrustes shape variables with 1,000 permutations (Adams & Collyer, 2016; Rohlf & Corti, 2000). For the modularity test, we combined the mandible and head dataset with normalize centroid size (Collyer et al., 2020) and considered the degree of modularity in two hypothesized modules (head and mandible) of Procrustes shape variables, comparing this to a null hypothesis of random assignment of variables, with 1,000 permutations (Adams, 2016).

We assessed the influence of the ecological predictors on the shape patterns using linear models of shape variation. The analyses were performed in R (R Development Core Team, v. 4.0.3, 2020) using the geomorph v. 3.3.2 and rrpp v. 0.6.2 packages (Collyer & Adams, 2020). We performed a Procrustes regression considering a residual randomization permutation procedure (Collyer et al. 2015) and contrasting the linear models, using the simple allometry as a null model. For this analysis, we employed three biological models with the null model nested with all the others. Additionally, we ran a model comparison based on log‐likelihoods for size and shape, including an adjusted tolerance for the shape models due to the number of variables exceeds the number of observations.

## 3 RESULTS

We found a strong allometric effect for both head (*p* = .001) and mandible shape (*p* = .001) using the combined dataset (major and minor workers), as well as for major and minor heads (*p* = .003 and *p* = .049, respectively), and minor mandibles (*p* = .002) when analyzed separately. Allometric trajectories of major and minor worker heads exhibit significant differences, with size accounting for 20% of the total shape variation (*p* = .001), subcastes explaining 15% (*p* = .001), and the interaction between size and subcaste describing 5% of the remaining variation (*p* = .006) (Table 1). Mandibles also show significant differences, in which size explains 28% (*p* = .001), subcastes represent 43% of shape variation (*p* = .001), whereas their interaction was not significant (Table 1). The homogeneity of slopes test found a significant (*p* = .006) difference between allometric slopes of the head, suggesting nonparallel slopes between majors and minors (Table 1). Additionally, this test indicated no significant (*p* = .067) difference between allometric slopes for the mandible, which implies parallel slopes (Table 1).

**TABLE 1:** Homogeneity of slopes test and Procrustes regression for head and mandibles allometric trajectories of majors and minor workers in *Pheidole*

Head	ResDf	RSS	SS	Rsq	F	Z	Pr(>F)
Common Allometry	51	0.213					
Group Allometries	50	0.197	0.016	0.049	4.073	2.253	**0.006**

	Df	SS	MS	Rsq	F	Z	Pr(>F)
Log (size)	1	0.068	0.068	0.205	17.217	4.424	**0.001**
Subcaste	1	0.049	0.049	0.149	12.529	3.824	**0.001**
Log (size): subcaste	1	0.016	0.016	0.049	4.073	2.253	**0.006**
Residuals	50	0.197	0.004	0.597			
Total	53	0.330					

Mandible	ResDf	RSS	SS	Rsq	F	Z	Pr(>F)
Common Allometry	51	0.294					
Group Allometries	50	0.283	0.011	0.011	1.944	1.566	0.067

	Df	SS	MS	Rsq	F	Z	Pr(>F)
Log (size)	1	0.283	0.283	0.280	50.014	3.285	**0.001**
Subcaste	1	0.433	0.433	0.429	76.626	3.995	**0.001**
Log (size): subcaste	1	0.011	0.011	0.011	1.944	1.566	0.067
Residuals	50	0.282	0.006	0.280			
Total	53	1.009					

Note

Bold values indicate statistical significance (*p* < .05).

**FIGURE 3:**
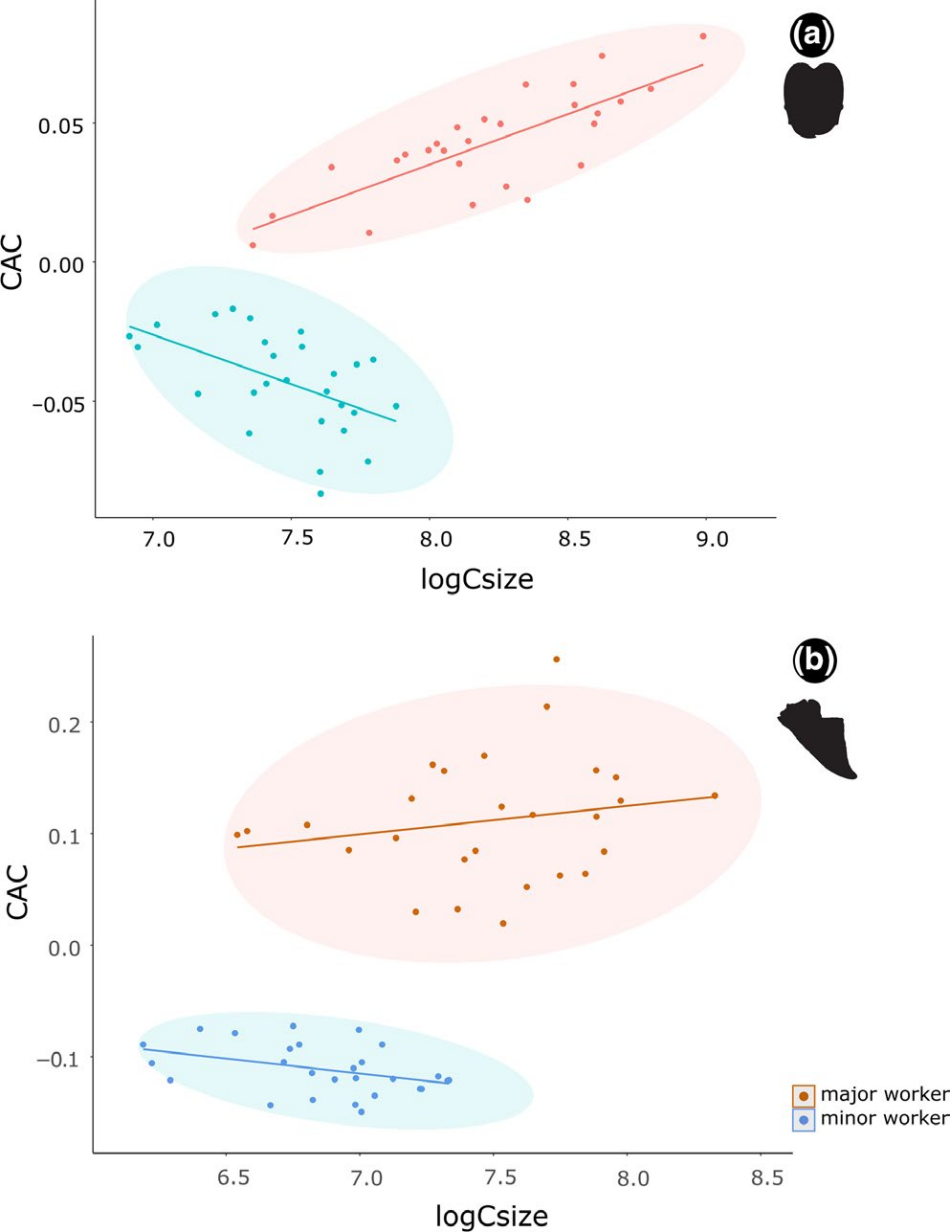
Head (a) and mandible (b) shape—size covariation plot based on common allometric component (CAC) analysis. Allometric trajectories colors denotes subcaste groups in *Pheidole* workers

The CAC plot for the head shows two separated slopes (majors and minors) with the common allometric component diverging dramatically with the size increasing, and with both majors and minors fitting closely to the regression vector (Figure 3b). Thin plate spline (TPS) deformations indicated head elongation for each size‐increasing unit, with subtle changes for minors when compared to majors. For the mandible, our plot also exhibits two separated slopes, in which CAC did not diverge as demonstrated for the head, but with most minors fitting closely to the common allometric regression vector, while majors are more dispersed around the regression vector (Figure 3a). Based on the TPS deformations, each increment of size unit is related to increases in the robustness of the mandible. Similar to the head pattern is observable for mandibles, in which minors tend to have subtle changes when compared to majors.

**FIGURE 4:**
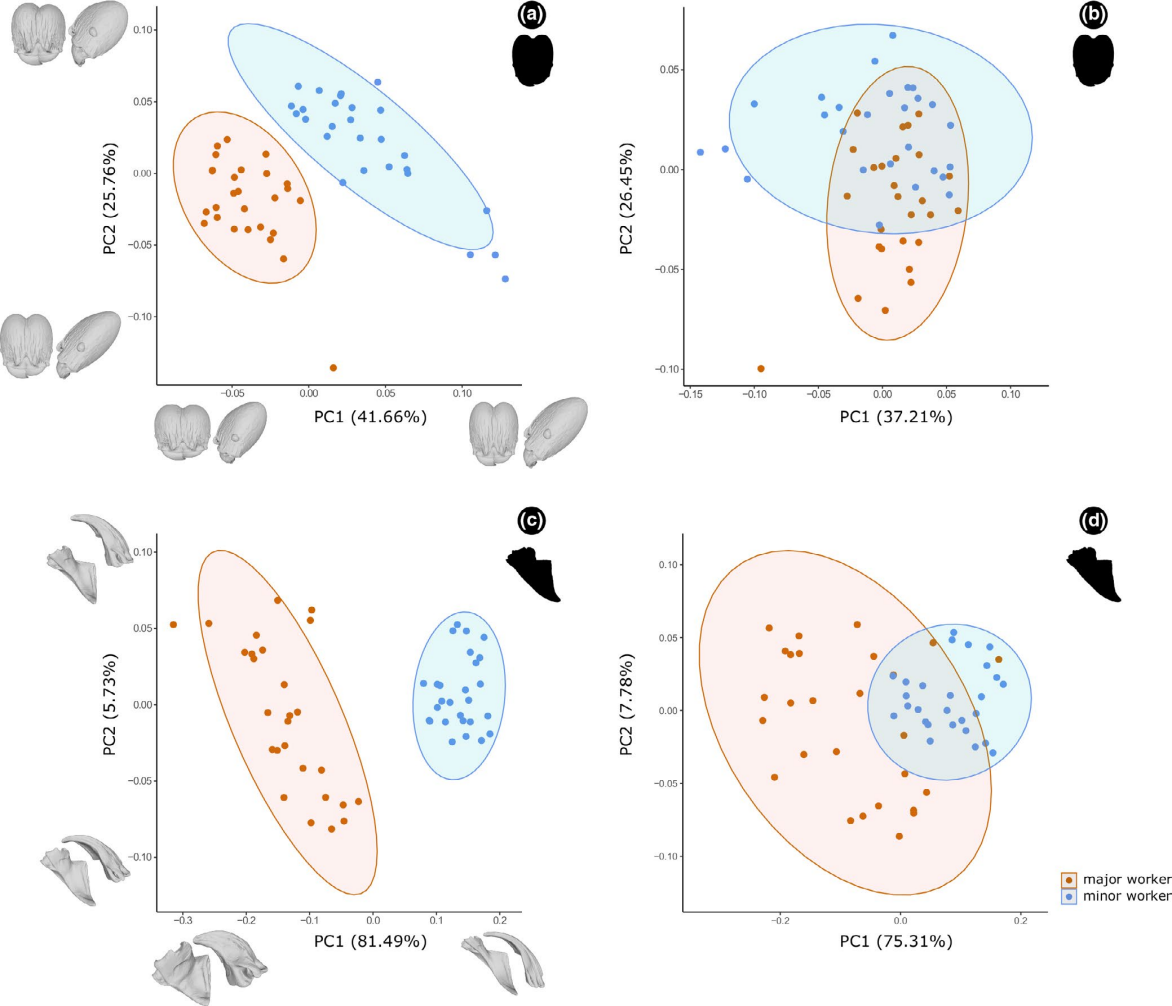
Principal component analysis of the head (a and b) and mandible (c and d) shape of *Pheidole* workers (a and c) with and (b and d) without allometric effects. Deformation models indicate extreme shapes along PC

All PCAs summarized shape variation in the head, with the first three PCs accounting for more than 70% of the observed variation (Figure 4a), while for mandible the first two PCs accounted for more than 87% of the variation (Figure 4c). In these PCAs, which simultaneously included major and minor workers, the main patterns of interspecific variation within and among subcastes can be visualized.

When not including correction for allometry, there was a clear separation between majors and minors on PC1 for both head and mandible (Figure 4a, c). Both tended to have either positive or negative scores in PC2 (Figure 4a, c). When the allometric effect was not accounted for, there was a considerable superposition of workers in morphospace, which was more evident for the head than the mandible (Figure 4b, d). The interpretation of the negative and positive scores was similar for PC1 and PC2 with an allometric effect. These first two allometry‐free PCs accounted for 37.21% and 26.45% of the variance for the head, and 75.31% and 7.78% for the mandible, respectively.

For the head, negative PC1 scores were related to short, wide, and dorsoventrally thick heads, whereas positive scores represented long, narrow, and dorsoventrally thin heads (Figure 4a). Positive PC2 scores corresponded partially to the elongation and thickness of the head, the convexity of the dorsal and ventral margins, as well as the vertexal lobe, with slightly curved margins and strongly projected lobe, whereas negative scores indicated strongly curved margins with slightly projected lobe (Figure 4a). In the case of the mandible, PC1 corresponded to slender mandibles in the positive scores, whereas negative scores indicated robust mandibles (Figure 4c). The PC2 described a short masticatory margin, with a slightly curved and elongated blade, and an obtuse angle between the mandible base and the internal margin, in the positive scores; whereas negative scores described a long masticatory margin, with a strongly curved and short blade, prominent apical tooth, and a straight angle between the mandible base and the internal margin (Figure 4b). Additionally, our results suggest that minor worker mandible (Figure 4c) and on a smaller scale the head (Figure 4a) are highly constrained in its morphological variation, while majors can diversify more in both structures (Figure 4a, c).

**FIGURE 5:**
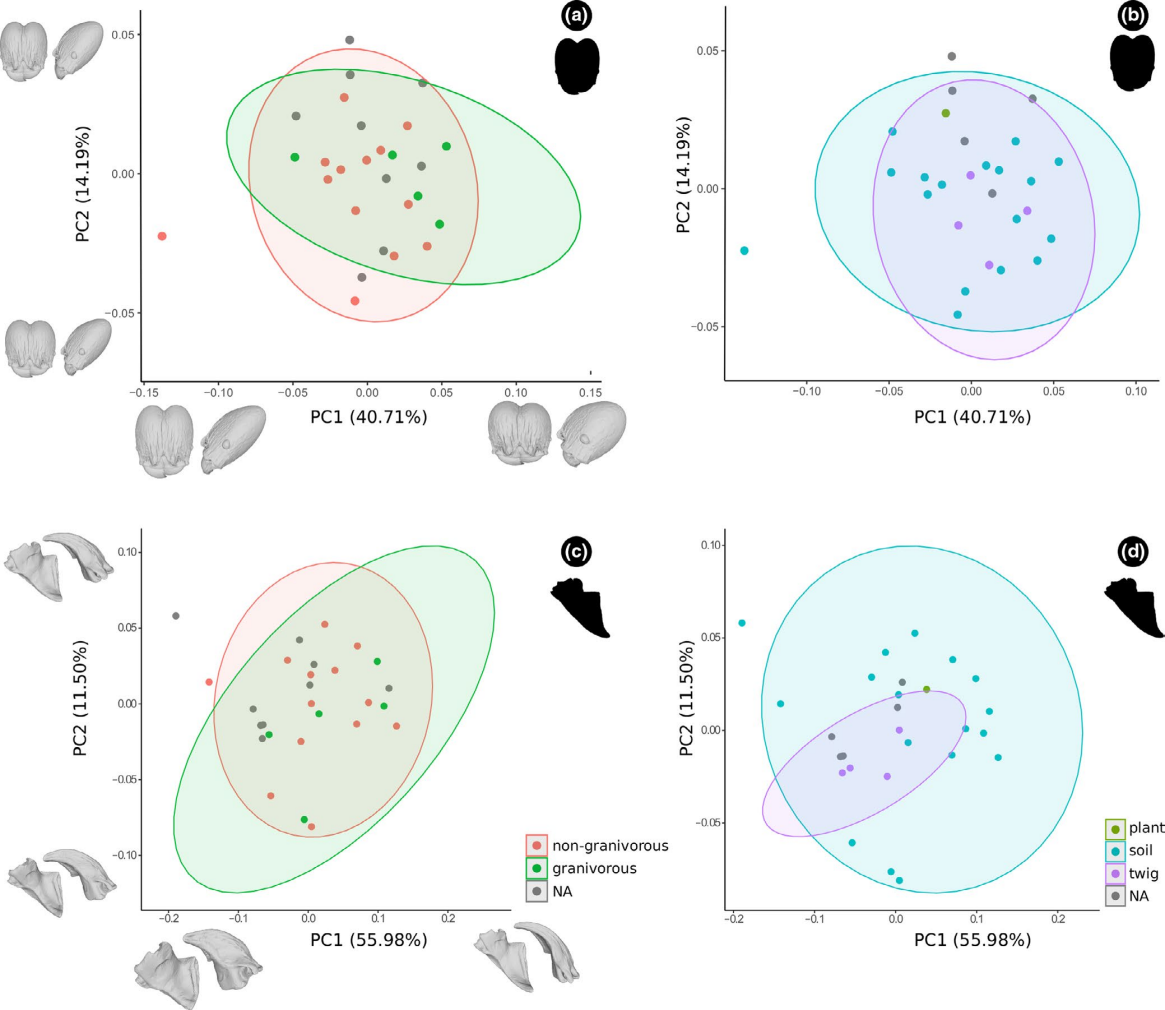
Principal component analysis of the head (a and b) and mandible (c and d) shape of *Pheidole* major workers considering (a and c) food preference and (b and d) nesting habitat. Deformation models indicate extreme shapes along PC

**FIGURE 6:**
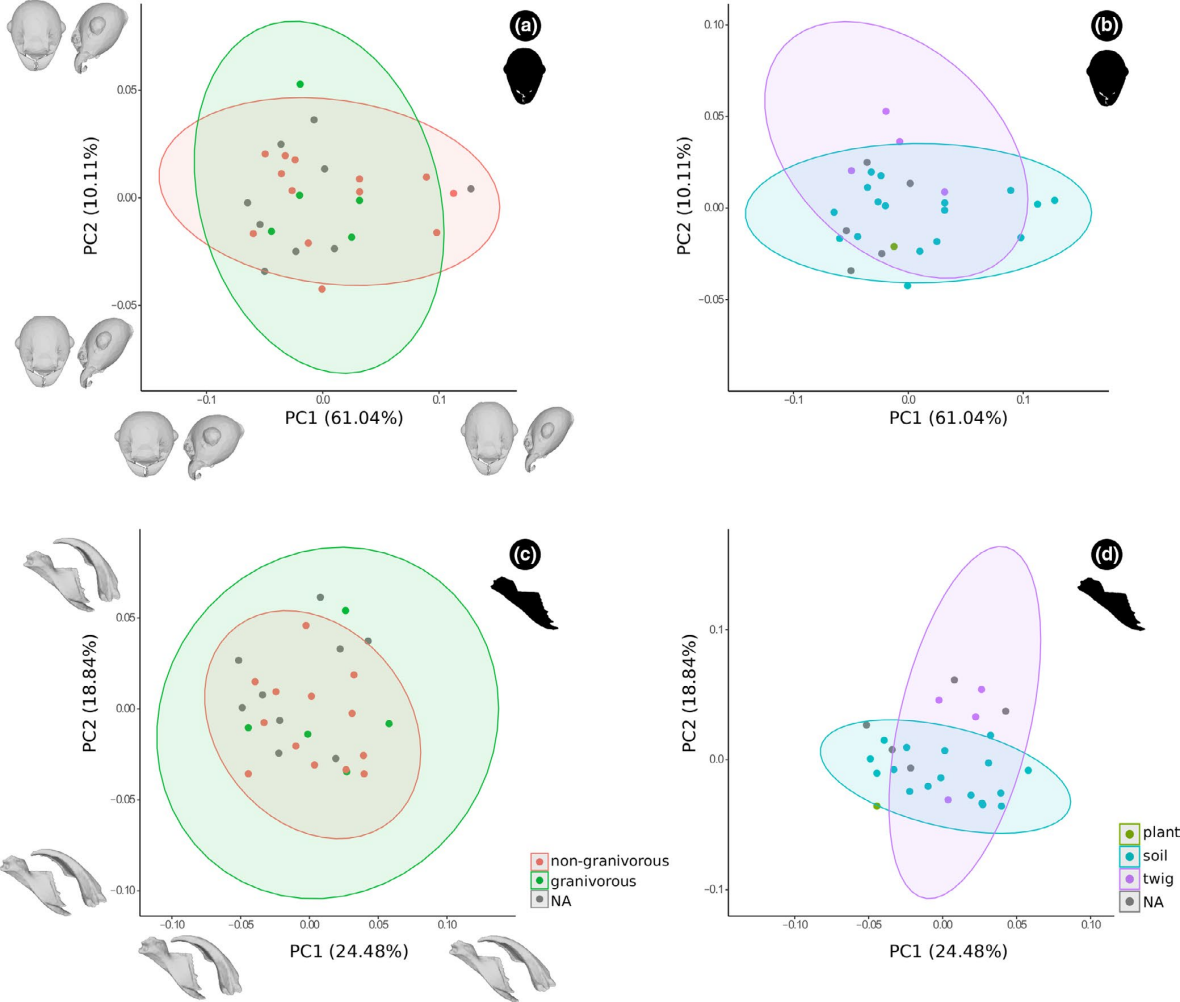
Principal component analysis of the head (a and b) and mandible (c and d) shape of *Pheidole* minor workers considering (a and c) food preference and (b and d) nesting habitat. Deformation models indicate extreme shapes along PC

In the noncombined dataset (Figures 5 and 6; S1 and S2), morphological interpretations of negative and positive scores in majors and minors were qualitatively similar to those found for the combined head dataset (Figure 4). However, the mandible results were slightly different, in which PC2 for majors described in the positive scores a slightly projected apical tooth, straight masticatory margin, and a wide mandibular base; whereas negative scores are related with a short apical tooth, inclined masticatory margin, and narrow mandibular base. For minors, it was necessary to assess the first four PCs to explain at least about 60% of the variation in the mandible (Figures 6; S2). The PC1 was represented for a narrow mandibular base and blade, slightly angulated masticatory margin, and with an obtuse angle between the mandibular base and the internal margin in the positive scores; while negative scores were associated with a broad mandibular base and blade, straight masticatory margin, and with a straight angle between the mandibular base and the internal margin. The PC2 described a long and strongly curved mandible apex, and an elongated ventral condyle in the positive scores, and negative scores associated with shorter and slightly curved mandible apex, and a shortened ventral condyle.

The reconstructed morphospaces (Figures 5 and 6) indicated an overlap in morphology among species with different food preferences, as well as nesting habits, with no statistically significant differences among these ecological predictor categories. This pattern suggests that variations in head and mandible for majors and minors could be relatively independent of ecological pressures. Procrustes analysis of variance showed that size is more important for mandible and head shape of majors (Z = 1.05 and 1.78, respectively; Table 2) and minor workers (Z = 2.43 and 1.44; Table 2) than the ecological predictors. However, size was the only statistically significant for major heads (*p* = .05; Table 2) and minor mandibles (*p* = .01; Table 2). Considering the effect of food preference and nesting habits on size, our results suggested no statistical significance (Table 2). But higher Z values were related to nesting for major mandible size (0.05; Table 2) and food preference for head size (0.71; Table 2); while minors presented higher values associated with food preference considering mandible (0.88; Table 2) and head sizes (0.80; Table 2).

**TABLE 2:** Results of Procrustes analysis of variance considering the head and mandible shape and size of *Pheidole* majors and minor workers

		Major worker					Minor worker				
		SS	Rsq	F	Z	Pr(>F)	SS	Rsq	F	Z	P(>F)
**Mandible Shape**	Size	0.02	0.07	1.76	1.05	0.13	0.01	0.10	2.71	2.43	**0.01**
	Food preference	0.04	0.14	1.61	1.00	0.11	0.01	0.08	1.11	0.43	0.34
	Nesting	0.02	0.06	0.69	−0.52	0.58	0.01	0.12	1.59	1.61	0.06
**Mandible Size**	Food preference	0.10	0.03	0.30	−0.50	0.73	0.35	0.15	1.93	0.88	0.20
	Nesting	0.37	0.09	0.74	0.05	0.54	0.43	0.19	1.58	0.75	0.23
**Head Shape**	Size	0.01	0.11	2.56	1.78	**0.05**	0.01	0.12	2.62	1.44	0.09
	Food preference	0.01	0.08	0.89	−0.15	0.55	0.01	0.04	0.46	−1.09	0.86
	Nesting	0.01	0.05	0.60	−0.95	0.82	0.01	0.08	0.89	0.01	0.47
**Head Size**	Food preference	0.45	0.13	1.55	0.71	0.25	0.21	0.14	1.71	0.80	0.22
	Nesting	0.33	0.10	0.76	0.04	0.55	0.21	0.15	1.18	0.47	0.35

Note

In the analysis of shape, the effect of size, food preference, and nesting were considered for head and mandible. In the analysis of size, only the effect of food preference and nesting were considered. Bold values indicate significant results.

The linear models show results congruent with those found in the morphospaces. Our results showed that the model including only size provided the best fit to our data (Tables 3 and 4). Additionally, for models involving ecological effects on size (Table 4), our results suggest that models including food preference has the best fit to our data. The only exception is for the minor mandible size, in which the model that includes only nesting habit has the best fit (Table 4). Conversely, majors showed a high Z value in the model that considered the influence of food preference on mandible (1.30; Table 3) and head (0.12; Table 3) shape; whereas minors show the highest Z value in the model that considered the nesting habit for mandible (2.25; Table 3) and head (0.48; Table 3).

**TABLE 3:** Results of model comparison using analysis of variance with randomization of residuals in a permutation procedure considering the head and mandible shape of *Pheidole* majors and minor workers

	Major worker					Minor worker				
	RSS	Rsq	*F*	*Z*	*P*(>*F*)	RSS	Rsq	*F*	*Z*	*P*(>*F*)
Mandible shape										
~size (null)	0.25	0.00				0.10	0.00			
~size + preference	0.21	0.14	1.89	1.30	0.11	0.09	0.08	1.07	0.34	0.37
~size + nesting	0.22	0.12	1.57	0.95	0.19	0.08	0.14	1.94	2.25	**0.01**
~size + preference + nesting	0.20	0.19	1.32	0.72	0.25	0.08	0.20	1.38	1.49	0.08
Head shape										
~size (null)	0.09	0.00				0.11	0.00			
~size + preference	0.08	0.07	0.98	0.12	0.44	0.10	0.05	0.60	−0.65	0.74
~size + nesting	0.08	0.06	0.86	−0.08	0.52	0.10	0.08	1.11	0.48	0.32
~size + preference + nesting	0.08	0.12	0.77	−0.60	0.71	0.10	0.03	0.27	0.15	0.45

Note

The null model used was the simple allometry model (shape ~ size), nested within all other models. For these models were considered the size, food preference, and nesting. Bold values indicate significant results.

**TABLE 4:** Results of model comparison using log‐likelihoods (with adjusted tolerance for shape data) considering head and mandible shape and size of *Pheidole* majors and minor workers

		Major worker		Minor worker	
		AIC (Allo)	AIC (Allo‐free)	AIC (Allo)	AIC (Allo‐free)
**Mandible Shape**	~size	**−1254.68**	**−1308.68**	**−3247.06**	**−3301.06**
	~preference	−1184.82	−1287.23	−3203.79	−3093.59
	~nesting	−1006.14	−1103.73	−3231.85	−3122.1
	~size + preference	−1182.34	−1240.34	−2989.14	−3047.14
	~size + nesting	−998.93	−1056.93	−3017.74	−3075.74
	~preference + nesting	−936.50	−1040.85	−2741.03	−2634.61
	~size + preference + nesting	−932.54	−994.536	−2526.78	−2588.78
**Mandible Size**	~preference	**32.17**	−	17.83	−
	~nesting	32.27	−	**17.11**	−
	~preference + nesting	35.53	−	19.86	−
**Head Shape**	~size	**−2189.83**	**−2243.83**	**−1583.99**	**−1637.99**
	~preference	−2122.59	−2230.17	−1117.88	−1412.65
	~nesting	−2122.46	−2229.29	−1331.57	−1427.51
	~size + preference	−2125.55	−2183.55	−1307.63	−1365.63
	~size + nesting	−2124.71	−2182.71	−1322.24	−1380.24
	~preference + nesting	−2062.16	−2171.85	−1054.82	−1155.24
	~size + preference + nesting	−2063.54	−2125.54	−1045.95	−1107.95
**Head Size**	~preference	**28.60**	−	**5.96**	−
	~nesting	28.71	−	6.47	−
	~preference + nesting	32.39	−	9.60	−

Note

For these models were considered the size, food preference, and nesting. Bold values indicate the lowest AIC values.

**FIGURE 7:**
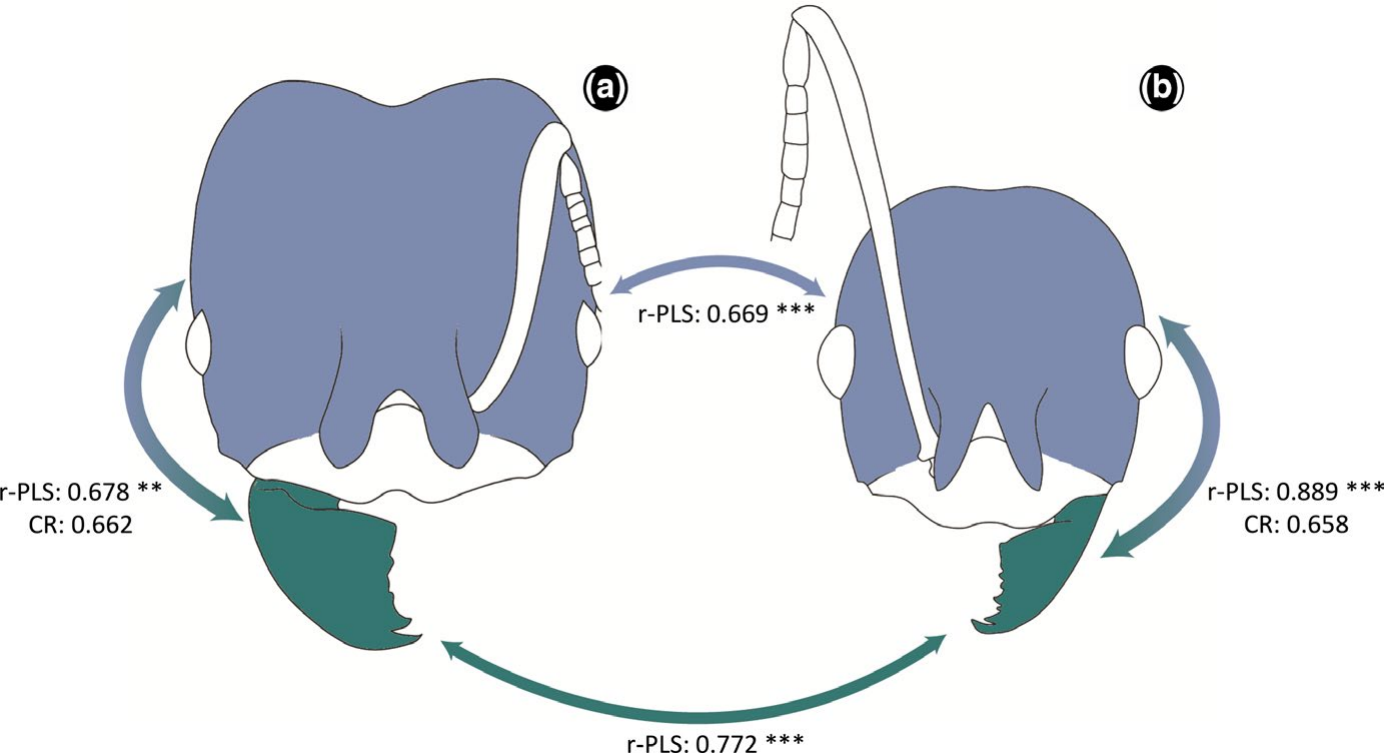
Results of integration (r‐PLS) and modularity (CR) tests for mandible and head of *Pheidole* workers; (a) major and (b) minor workers. The arrows indicate the modules and its respective values

Regarding morphological integration, our results indicated significant scores with high r‐PLS values for all combinations between and within majors and minor workers, and between their mandible and head (Figure 7). This result implies that morphological changes in the mandible were accompanied by corresponding changes in the head. The higher r‐PLS results were related to the minor head and mandible (r‐PLS = 0.889; effect size = 4.3796), and between majors and minor worker's mandibles (r‐PLS = 0.772; effect size = 2.7842). As an assessment of the modularity of these structures, the observed covariance ratio (CR) coefficient was significantly lower (majors CR = 0.662, *p* =.001, effect size = −19.8179; and minors CR: 0.658, *p* = .001, effect size = −18.9565; Figure 7), suggesting that there was a strong degree of independence between the two modules. When allometry was not accounted for, integration and modularity results were qualitatively similar, showing the same patterns with slightly higher values.

## 4 DISCUSSION

Changes related to size and shape, their evolutionary history, and the effect of ecological predictors driving those changes are a cornerstone of macroecology and macroevolution (Bright et al., 2016; Dumont et al., 2011; Olsen, 2017). In ants, workers of about 13% of the species are subdivided into morphologically variable and ecologically adapted subcastes (Oster & Wilson, 1978), so understanding this pattern and how they are influenced by biotic and abiotic predictors is a key concern in myrmecology. Several studies have explored general morphological consequences of this division into subcastes and their morphological adaptations (Holley et al., 2016; Powell, 2008, 2009; Powell & Franks, 2005, 2006; Tschá & Pie, 2019). However, with new sources of information, such as microCT, new questions can be postulated considering body parts that were not previously explored, due to constraints in the quantification of their size and/or complexity.

Here, we used a comparative approach to explore how the head and mandible shape of workers vary and are affected by allometry, modularity, and integration in *Pheidole*, while also describing their morphospace. Mouthparts are often viewed as one of the targets for natural selection, which is also expected considering the ecological demands associated with the division of labour between *Pheidole* majors and minor workers. This is also true for the head, as this body region holds the muscles and the appendages related to the feeding and colony maintenance, as well as the sense organs. Contrary to the belief about ecological pressures over mandible and head shape, we find that, in *Pheidole*, morphological variation of these structures is characterized by a strong degree of independence between them coupled to high integration and a pronounced allometric effect, with little correlation with food preferences and nesting habits.

Our integration results indicate that there is greater variation in the head shape of major workers, which would not be directly associated with the mandibular demands (Figure 7). The strong degree of independence between head and mandible for majors and minors suggests that these structures display significant modularity when compared to the null hypothesis of no modular association (Adams, 2016). Strong integration and significant modularity could represent a key factor in shaping the morphology and diversity of heads and mandibles in *Pheidole* workers, thus constraining the possible morphological patterns that these structures can assume, in spite of ecological pressures.

We found a strong relationship between head and mandible with size, both for minor workers and head shape in majors. This suggests that size is an effective mechanism by which *Pheidole* workers may adjust to biological demands (*e*.*g*., changes in food preference and nesting habits). Our analysis also showed that, although there is a significant change in shape related to size within the worker caste, allometry does not explain all shape variation (Table 1). Differences between subcastes had a considerably high effect on the head shape, but with size explaining most of the variation, whereas for the mandible shape, the allometric effect was significantly lower than the subcaste (Table 1). These results suggest that majors and minor workers are more different than expected only due to size variation, which implies that morphological changes may be largely related to different tasks that each subcaste performs within the colony. These results corroborate Pie and Traniello (2007), who argued that worker shape in *Pheidole* varies predominantly through changes in size, even considering that this genus inhabits diverse ecological niches. Analysis of common allometric component (CAC) showed that size‐related shape changes are mostly concentrated on the elongation of the head and thickness of the mandible. These results described a contrast between small individuals with relatively square‐shape heads and slender mandibles, while large individuals present comparatively elongated heads and robust mandibles. Here, mandible shows parallel trajectories between majors and minor workers and head presents divergent trajectories, with majors exhibiting more positive slopes relative to minors. This result suggests that majors have higher rates of shape change relative to size than do minor workers when the head is considered, while mandible has a consistent rate between majors and minors. This pattern is largely consistent with what we find in the reconstruction of morphospace occupied by majors and minor workers (Figure 4; see discussion below).

The indication that food preference and nesting habit may not explain variation in the head and mandible shape, mostly considering the granivorous habits in *Pheidole*, is consistent with some previous publications that used distinct measurements and analysis (*i*.*e*., Holley et al., 2016). In this sense, the muscle demands would not influence the head shape of seed‐harvesting species, nor its size, as previously shown by Holley et al., (2016). Wheeler (1910) already hypothesized the absence of a direct relationship between head shape and granivory, arguing that morphological modifications related to the seed processing would also be needed to tear exoskeletons from other insects commonly collected by these ants. Indeed, our results indicate that potentially not only head shape is not affected by these requirements, including nesting habit, but also that this seems to be the case for mandible shape, in which distinct morphologies that occupy morphospace would compensate for pressures related to these ecological predictors. An alternative possibility might be related to behavioral or physiological aspects, which in this case would facilitate the exploration of different niches, regardless of the head and mandible shapes.

These results should be taken with the caveat that we did not consider these correlations in a phylogenetic context, and the sample size of species was relatively small. However, the main risk of an unmeasured phylogenetic effect would be a spurious ecology‐morphology correlation, not a lack of correlation, so we do not think it likely that consideration of phylogeny will qualitatively change our results. To test this expectation, a more comprehensive study including a more comprehensive number of species from different biogeographical regions and with reliable biological and molecular data is necessary.

Aside from the morphological variation in the mandible shape for majors and minor workers regarding thickness, and continuous variation from robust mandibles to slender ones, and continuous variation in the curvature, our results showed an important variation related to the mandibular base. This region, which is often seen as more conserved, exhibited a set of key modifications such as its angle to the internal margin, the size, and shape of the atala (Richter et al., 2020), as well as the internal articulation margin. These changes are related to the muscle insertions (*i*.*e*., atala with the opening muscles) and points of articulation with the head. Variations in those regions would be directly associated with changes in the way these ants use their mandibles to interact with the environment; however, more data considering behavior and biomechanical properties are necessary to elucidate their functionality. Additionally, major workers exhibited more variation in the elongation of the mandible, as well changes in its thickness (PC1 ‐ Figure 7a, b), when compared to the minors (PC1 ‐ Figure 7c, d).

Our results also suggest that variation is much stronger in major worker mandibles than in minors (Figure 4c). Cases of dimorphism among workers are particularly unique, especially considering its impact on the division of labour in the colony. In this process, as the minor workers provide all the essential and basic daily functions of the colony, majors can specialize without compromising colony‐level ergonomic efficiency. Other cases have similar patterns, in which the structures released from their original functions can vary and diversify without compromising the system. Classic examples of this are the cichlid pharyngeal jaws and the evolution of new genes. In African cichlid fishes, the explosive evolution in several species with morphological highly specialized oral jaws was mainly caused by the presence of a pharyngeal jaw (Albertson et al., 2005; Burress et al., 2019; Kocher et al., 2004). For these fishes, the pharyngeal jaw, as it plays a role in the basic chewing functions, enabled the mandible to diversify into several different morphological patterns (Albertson et al., 2005; Burress et al., 2019; Kocher et al., 2004; Liem, 1973; Liem & Osse, 1975). The same occurs in the process of evolution of new genes, in which gene duplication, seen as one of the most important contributors to this process, allows new copies to mutate, as their functions will not be lost due to its duplication (Kaessman, 2010; Long et al., 2003, 2013; Taylor & Raes, 2004).

Considering head morphology, contrary to that found for mandible variation, minor workers head diversified along different shape axes (Figure 6c, d), with PC1 being more important for minors than for majors (Figure 6a, b). Regarding minor workers, most of the variation in head shape was related to elongation and thickening. This enables a significant increase in muscle adhesion surface, as well its size, consequently promoting growth in its volume. Additionally, head diversification in minor workers is slightly constrained in its morphospace, when compared with majors, following a similar pattern as that suggested for mandible diversification in our results. As discussed, this higher volume may not be related to specific food preferences, either nesting habit. However, it could enable a considerable advantage for defense, food loading, as well as the processing of a variety of food items (*e.g.,* Wheeler, 1910).

Wilson (2003) has postulated that *Pheidole's* hyperdiversity may result from the dimorphism between its workers, the majors being specialized individuals related to specific tasks such as defense, and food transport and processing, as well as assuming distinct functions in the absence of minors. On the other hand, Mertl el al. (2010) proposed that the occupation of niches in *Pheidole* would have as its main cause behavior‐related adaptations, and morphological modifications would be less involved in its speciation. Our results demonstrate that our tested ecological predictors may not drive the morphological changes expressed by *Pheidole*, suggesting that morphological integration, modularity, and allometry are the determinant attributes. The behavioral flexibility provided by a dimorphic worker caste could have buffered selective pressures that would have otherwise lead to morphological specialization. However, little is known about how the structural developmental constraints that influenced the diversification of this genus, especially considering biomechanical aspects, as well as behavioral predictors. The reasons for the hyperdiversity in *Pheidole* remain unknown; however, the present results addressed an important piece on this puzzle.

## CONFLICT OF INTEREST

None declared.

## AUTHOR CONTRIBUTION

**Alexandre Casadei Ferreira:** Conceptualization (lead); Data curation (lead); Formal analysis (lead); Funding acquisition (equal); Investigation (lead); Methodology (equal); Project administration (equal); Software (equal); Validation (equal); Visualization (lead); Writing‐original draft (lead); Writing‐review & editing (equal). **Nicholas R. Friedman:** Conceptualization (supporting); Formal analysis (supporting); Investigation (supporting); Methodology (equal); Software (equal); Validation (equal); Visualization (equal); Writing‐review & editing (equal). **Evan P. Economo:** Conceptualization (equal); Formal analysis (supporting); Funding acquisition (equal); Investigation (supporting); Methodology (supporting); Resources (equal); Software (equal); Supervision (equal); Validation (equal); Visualization (equal); Writing‐review & editing (equal). **Marcio R. Pie:** Conceptualization (equal); Formal analysis (supporting); Investigation (equal); Methodology (equal); Resources (equal); Supervision (equal); Validation (equal); Visualization (equal); Writing‐review & editing (equal). **Rodrigo Feitosa:** Conceptualization (equal); Funding acquisition (equal); Investigation (equal); Project administration (equal); Resources (equal); Supervision (lead); Validation (equal); Visualization (equal); Writing‐review & editing (equal).

## Supporting information

Figure S1

Figure S2

Table S1

## Data Availability

Data for this paper can be accessed on Dryad at https://doi.org/10.5061/dryad.1rn8pk0sz.
