# Supplementary material for: Head and mandible shapes are highly integrated yet represent two distinct modules within and among worker subcastes of the ant genus Pheidole
Source: Ecol Evol. 2021 May 1;11(11):6104–18. doi: 10.1002/ece3.7422 (PMC8207162; doi:10.1002/ece3.7422)
Supplement: Supplementary file 1 — Figure S1 [file ECE3-11-6104-s001.docx]

**
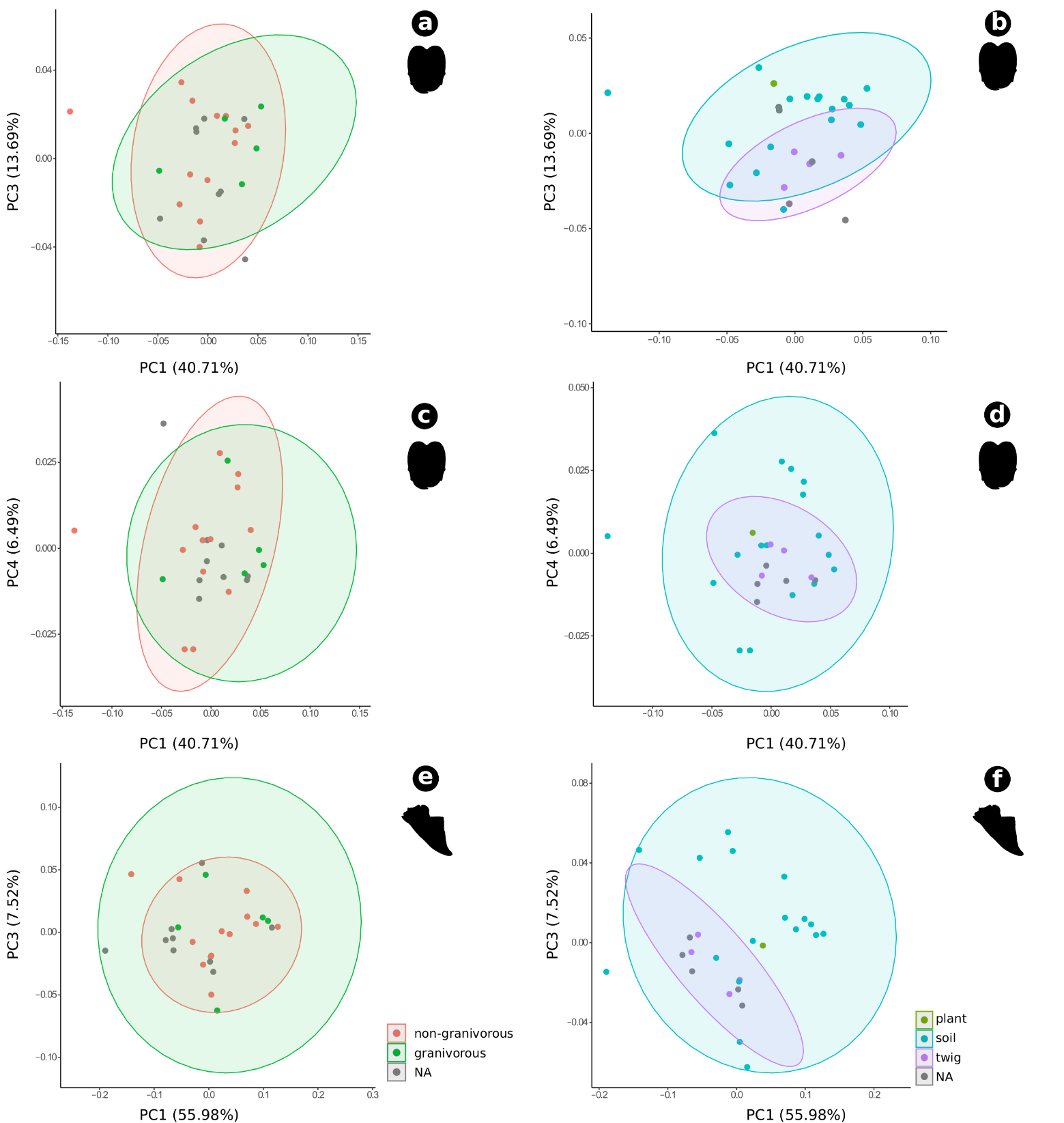
**

**Figure S1**. Principal component analysis of the head (a, b, c, and d), considering PC3 and PC4, and mandible (e and f) shape, considering PC3, of *Pheidole* major workers.
