# Supplementary material for: Head and mandible shapes are highly integrated yet represent two distinct modules within and among worker subcastes of the ant genus Pheidole
Source: Ecol Evol. 2021 May 1;11(11):6104–18. doi: 10.1002/ece3.7422 (PMC8207162; doi:10.1002/ece3.7422)
Supplement: Supplementary file 2 — Figure S2 [file ECE3-11-6104-s003.docx]

**
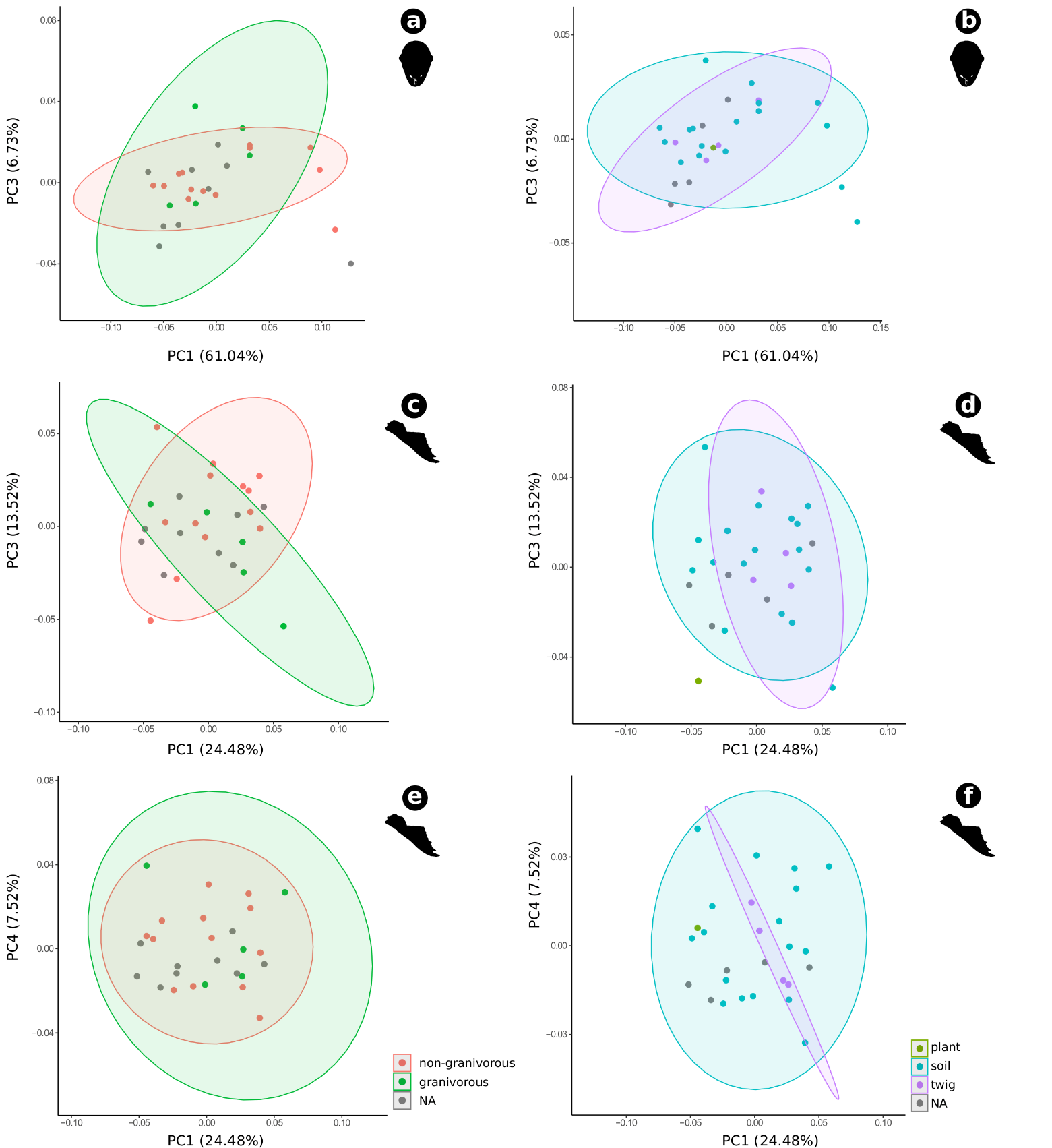
**

**Figure S2**. Principal component analysis of the head (a and b), considering PC3, and mandible (c, d, e, and f) shape, considering PC3 and PC4, of *Pheidole* minor workers.
