# Supplementary material for: Head and mandible shapes are highly integrated yet represent two distinct modules within and among worker subcastes of the ant genus Pheidole
Source: Ecol Evol. 2021 May 1;11(11):6104–18. doi: 10.1002/ece3.7422 (PMC8207162; doi:10.1002/ece3.7422)
Supplement: Supplementary file 3 — Table S1 [file ECE3-11-6104-s002.docx]

| **Species** | **Food Preference** | **Nesting** | **Source** |
| --- | --- | --- | --- |
| *Pheidole aberrans* | Unknown | Soil | Literature |
| *Pheidole* acf017 | Granivorous | Soil | Personal observation |
| *Pheidole* acf051 | Unknown | Unknown | - |
| *Pheidole* aff. *hetschkoi* | Unknown | Unknown | - |
| *Pheidole* aff. *lancifera* | Unknown | Soil | Personal observation |
| *Pheidole* aff. *lucretii* | Non-granivorous | Soil | Personal observation |
| *Pheidole aper* | Non-granivorous | Twig | Literature |
| *Pheidole astur* | Unknown | Soil | Literature |
| *Pheidole cavifrons* | Non-granivorous | Soil | Literature |
| *Pheidole cursor* | Non-granivorous | Soil | Literature |
| *Pheidole deima* | Non-granivorous | Soil | Personal observation |
| *Pheidole dyctiota* | Unknown | Twig | Personal observation |
| *Pheidole fimbriata* | Non-granivorous | Soil | Literature |
| *Pheidole gibba* | Non-granivorous | Twig | Personal observation |
| *Pheidole gigaflavens* | Non-granivorous | Soil | Literature |
| *Pheidole guilelmimuelleri* | Granivorous | Twig | Personal observation |
| *Pheidole heyeri* | Unknown | Twig | Personal observation |
| *Pheidole lucretii* | Non-granivorous | Soil | Personal observation |
| *Pheidole minutula* | Non-granivorous | Plant | Literature |
| *Pheidole obapara* | Unknown | Twig | Personal observation |
| *Pheidole obtusospinosa* | Granivorous | Soil | Literature |
| *Pheidole pedana* | Unknown | Unknown | - |
| *Pheidole rhea* | Granivorous | Soil | Literature |
| *Pheidole scolioceps* | Non-granivorous | Soil | Literature |
| *Pheidole subarmata* | Granivorous | Soil | Literature |
| *Pheidole valens* | Non-granivorous | Soil | Personal observation |
| *Pheidole vallifica* | Non-granivorous | Soil | Literature |

**Table S1** Species scanned including data regarding food preference and nesting habitat and their source.
